# Management by Committee

**Published:** 1912-03-23

**Authors:** 


### MANAGEMENT BY COMMITTEE.

It is rare indeed to find nowadays a hospital where its affairs are managed on narrow personal lines, as was the case formerly at many hospitals. The reason for this is no doubt largely due to the personal interest taken in the administration of hospitals, and all that concerns their efficient management and the welfare of their inmates, by members of the Royal Family. This great change began to prove effective from the moment the late King Edward VII. when Prince of Wales took up these questions, studied them, and heartily threw himself into the work of reform. He rejoiced to find a field where he could devote his energies usefully to the solving of many problems and the wise removal of defects which impeded or delayed progress in the hospital world. By personal service, by wise counsel, and by an interest both deep and genuine, King Edward's connection with hospitals will go down to history as one of the most important and memorable proofs, in comparatively recent times, of the enormous developments for good in our social system. King Edward may be said to have done for hospital management, by the introduction of a high standard of personal conduct on the part of everybody responsible for the management and work of institutions for the sick, what Lord Lister did by his personal devotion and example in connection with the scientific treatment of disease. A wholesome atmosphere of freedom from parasites of all descriptions, and a standard of the highest personal conduct, have attracted to the hospital field, as workers, many of the very best men and women of our day and generation. They have helped to make our voluntary institutions the glory of the nation and the wonder of the world.

It is not easy, though it is exceedingly instructive and helpful, to find in the present day examples of the old faulty systems of government by committee. Two such instances have, however, during the last twelve months been brought prominently to the notice of the profession and hospital workers at Chester and at Spalding. At Chester the persecution of Miss Addis, the matron, who for nine consecutive years rendered excellent service there and whose character is beyond reproach, has revealed some serious evils which may result from the absence of a controlling mind of great intelligence and the highest standard of personal conduct amongst those responsible for the administration of a voluntary hospital even in the present day. At Spalding the management of the Johnson Hospital was first questioned by the fifty-seventh of the series of expert and impartial reports we are publishing on our voluntary hospitals, which appeared in The Hospital of November 18, 1911. This report produced a public inquiry held by the Charity Commissioners, and the full account of its proceedings will be found in the *Spalding Guardian* of the 2nd instant and in the *Lincolnshire, Boston, and Spalding Free Press* of the 5th instant. No hospital manager who takes a real interest in his work should miss the study of these reports of the proceedings before the Charity Commissioners in conjunction with our reports of November 18 and December 2, 1911.

